# What is the correct scientific name for “Fuling” medicinal mushroom?

**DOI:** 10.1080/21501203.2022.2089755

**Published:** 2022-06-20

**Authors:** Viktor Papp, Yu-Cheng Dai

**Affiliations:** a Department of Botany, Hungarian University of Agriculture and Life Sciences, Budapest, Hungary; b Institute of Microbiology, School of Ecology and Nature Conservation, Beijing Forestry University, Beijing, China

**Keywords:** *Poria*, *Wolfiporia*, *Pachyma*, *Hoelen*, taxonomy, nomenclature

## Abstract

In recent years, the scientific names of many cultivated and well-known medicinal fungal species have been changed. However, the results of taxonomic and nomenclature works on these economically important fungi are often overlooked or ignored in applied researches. The incorrect use of scientific names may cause uncertainty in research and in the global medicinal mushroom market. In this paper, we briefly review the current taxonomy and nomenclature of “Fuling” medicinal mushroom and make a proposal for biochemists, pharmacists and businessmen on the correct use of scientific names related to this species. Based on the recent taxonomic results and nomenclatural proposals, the use of the names *Wolfiporia extensa, W. cocos* and especially *Poria cocos* for the “Fuling” mushroom are incorrect and misleading; therefore, the acceptance of the names *Pachyma hoelen* or *Wolfiporia hoelen* is recommended.

## 1. Introduction

The use of correct scientific name for medicinal mushroom species is crucial for the traditional Chinese medicinal studies and industries of macrofungi (Zhou 2019). However, the results of taxonomic and nomenclature works on these economically important fungi are often overlooked or ignored in applied researches (e.g. Papp et al. 2017; Shen et al. 2021). The continued use of popular names that is scientifically incorrect may also be due to the difficulties to survey the sometimes conflicting literatures, especially for researchers less experienced in taxonomy and nomenclature. Incorrect scientific names are used for various reasons: (1) use a synonym name which has no priority, but taxonomically correct, e.g. use the name *Coriolus versicolor* (homotypic synonym) instead of *Trametes versicolor* or use the name *Pholiota nameko* (heterotypic synonym) instead of *Ph. microspora* (Neda 2008; Justo and Hibbett 2011; Lee et al. 2020); (2) use a name which is taxonomically represent the same species, but nomenclaturally not validly published, e.g. use the name *Ganoderma cupreolaccatum* instead of *G. pfeifferi* (Papp et al. 2022); (3) use a synonym name which systematically incorrect, because generic name has been represent a different group of fungi, e.g. use the name *Antrodia camphorata* instead of *Taiwanofungus camphoratus* or use the name *Lentinus edodes* instead of *Lentinula edodes* (Wu et al. 2018; Menolli Jr. et al. 2022); (4) use a name which published validly, but represent a different species, e.g. use the name *Ganoderma lucidum* for Lingzhi or *Phellinus linteus* for Sanghuang mushroom (Cao et al. 2012; Zhou et al. 2016; Dai et al. 2017). The latter misinterpretation causes the most difficulties; in addition to nomenclatural confusion, it hinders or significantly complicates orientation in scientific literatures due to the uncertainty of the examined species.

In recent years, the scientific names of many cultivated and well-known medicinal fungal species have been changed, due to the use of molecular genetic methods. These integrative taxonomic works have shown that several well-known and widely cultivated medicinal mushroom species in East Asia, e.g. in *Auricularia, Flammulina, Ganoderma*, etc. are not identical to the species introduced from Europe or North America (Cao et al. 2012; Wu et al. 2014; Wang et al. 2018, 2021). Recently, the “Fuling” mushroom was also studied by Wu et al. (2020) for this purpose. In this paper, we briefly review the current taxonomy and nomenclature of this important fungal species and make a proposal for biochemists, pharmacists and businessmen on the correct use of scientific names related to the “Fuling” mushroom.

## 2. Differences between “Fuling” and “Tuckahoe” samples

The examination of the taxonomic differences between the North American “Tuckahoe” fungus and the “Fuling” known from Asia is not a newfangled topic, since two centuries ago Fries (1822) described them as two separate species. The first valid scientific name of the fungal sclerotia of Tuckahoe was *Sclerotium cocos* Schwein. Fries (1822) accepted this name in his new genus *Pachyma* Fr. and distinguished the species *P. hoelen* Fr., which he characterised as a little-known medicinal species from China. Based on the examination of a Chinese specimen received from a drug store, W. A. Murrill was also stated that *P. hoelen* is distinct from *P. cocos* Fr. (Merrill 1917). Recently, *P. hoelen* was neotypified by Wu et al. (2020), who carried out a single-locus (ITS) and multigene (ITS, LSU, *tef1, rpb2*) phylogeny, and confirmed that *P. hoelen*, which is widely cultivated in China, is not conspecific with the North American *P. cocos* (syn. *Wolfiporia cocos* (F.A. Wolf) Ryvarden & Gilb.). Based on the comparison of OrthoMCL gene families and whole genomes, genetic differences were also found by Luo et al. (2020) between North American (MD-104, see Floudas et al. 2012) and Chinese (CGMCC5.78) strains. Similar result was obtained by Cao et al. (2021), who sequenced a cultivated strain of Fuling mushroom (2018LT001), originated from Hubei, China.

Consequently, both traditional morphological examinations (Fries 1822; Merrill 1917), molecular phylogenetic studies based on barcoding sequences (Wu et al. 2020), and genome-level comparisons (Luo et al. 2020; Cao et al. 2021) prove that Asian Fuling samples differs from the North Americans. So, it seems justified to treat “Fuling” (*Pachyma hoelen*) and *Sclerotium cocos* (syn. *Wolfiporia cocos*) as a separate species.

## 3. What is the most popular scientific name for “Fuling”?

**Figure 1.**
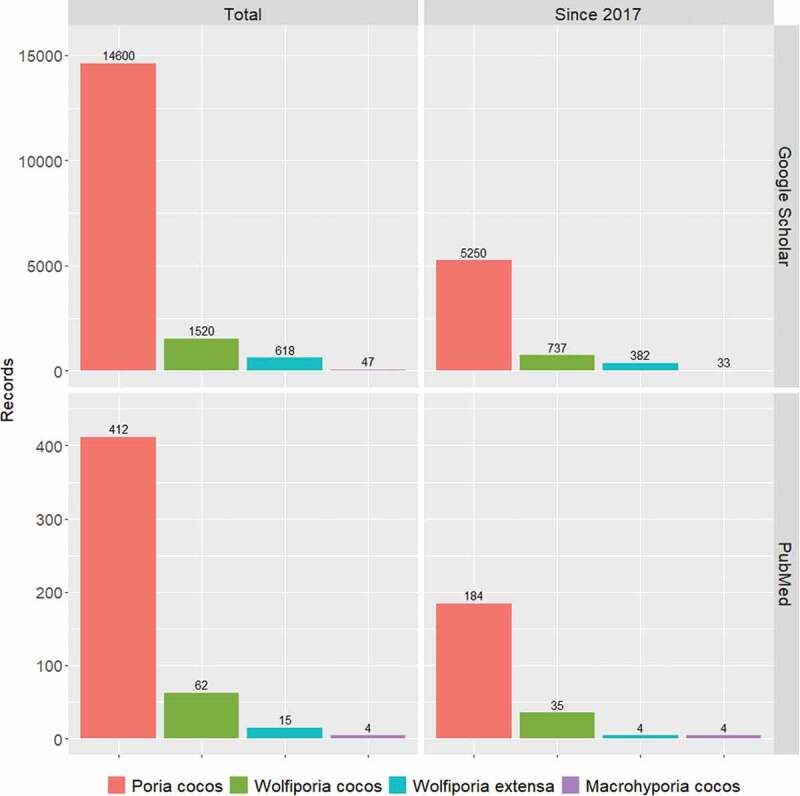
The popularity of scientific names related to “Fuling” mushroom based on the PubMed and Google Scholar databases. In the PubMed database, the search was based on title/abstract; and for the name “*Poria cocos*”, the following criterion was used: (“poria cocos” [Title/Abstract]) NOT (wolfiporia [Title/Abstract]).

In the case of economically important fungal species, it is more difficult for the market and thus the applied research to apply the changes in scientific names. This was also the case with the “Fuling” mushroom. Although the economically significant form of “Fuling” is the sclerotium (asexual form), the name given to the species is based on its sexual form. The first of these names was *Poria cocos* F.A. Wolf, which was proposed by Wolf (1922) based on a sample collected from North Carolina, United States. This binom has been the most widely used scientific name since, and the nomenclature changes proposed over the past 100 years have not been able to change it. The name *P. cocos* is used more widely than *Wolfiporia cocos* and all other related names based on PubMed (PM) and Google Scholar (GS) searches, respectively. The data show that the name “*Poria cocos*” was used 6.65 times more than “*Wolfporia cocos*” on PM, and 9.6 times more on GS (Figure 1). Comparing the total number of records with the records of the last 5 years, we see that “*Wolfporia cocos*” is getting to be slightly more popular, but “*Poria cocos*” is still highly preferred (used 5.25 and 7.12 times more often on PM and GS, respectively). Thus, the taxonomic changes in the past 100 years have not had a breakthrough effect either, as the widely known name *Wolfiporia cocos* (Ryvarden and Gilbertson 1984) is also in less than one-fifth of the results. The homotypic synonym *Macrohyporia cocos* (Johansen and Ryvarden 1979) and the name *M. extensa* and *Wolfiporia extensa* (bas. *Daedalea extensa* Peck) suggested as an older heterotypic synonym of *W. cocos* (Ginns and Lowe 1983; Ginns 1984) had remained obscure. Given that the concept of One Fungus-One Name (1F1N) was only introduced in the *Melbourne Code* (McNeill et al. 2012), the names that preceded *Poria cocos*, based on the economically significant asexual form and are now available (e.g. *Pachyma* spp.), have not been used so far, despite their nomenclature priority (Wu et al. 2020).

## 4. Why the scientific name *Poria cocos* is not applicable for “Fuling”?

Although *Poria cocos* is a long and widely used name as a scientific binomial, its application for the “Fuling” mushroom is not possible from a taxonomic as well as nomenclature point of view. Nomenclature and taxonomy intersect at the type (specimen or type species), as designated through the ICN to anchor scientific names to the taxonomical concepts. The type of the *Poria* Pers. genus is *P. medulla-panis* (Jacq.) Cooke, which recently belongs to the genus *Perenniporia* Murrill (Decock and Stalpers 2006). This species is systematically very far from “Fuling” mushroom (Justo et al. 2017), so the generic name *Poria* is taxonomically not applicable to this species. Furthermore, from a nomenclature point of view, *Poria* Pers. is illegitimate (ICN, Art. 53.1.). In the case of *Poria cocos*, not only the generic name is problematic, as this name and species are related to type specimen originated from North America. So, if there is a species-level difference between the North American (“Tuckahoe”) and Asian (“Fuling”) samples, another epithet should be given for the latter.

## 5. Which scientific name should be used for “Fuling”?

To give the correct scientific name for “Fuling”, in addition to clarifying taxonomic issues, it is further complicated that this fungus also has a sexual and asexual form that was previously given separate names. Based on the changes in Art. 59 of the ICN (Turland et al. 2018), the legitimate generic names typified by asexual fungal stages are treated equally for the purposes of establishing priority. Based on this, Wu et al. (2020) concluded that the valid name for “Fuling” is *Pachyma hoelen* Fr. Recently, the competing sexual-asexual generic names in *Agaricomycotina* (*Basidiomycota*) were evaluated by Stalpers et al. (2021), and suggested to protect the sexual name *Wolfiporia*. This is justified that *Wolfiporia* Ryvarden & Gilb. is more widely used than *Pachyma* and other related, but taxonomically uncertain, asexual generic names (viz. *Gemmularia* Raf. and *Tucahus* Raf.). If the proposal by Stalpers et al. (2021) will be approved by the Nomenclature Committee for Fungi, the correct scientific name for “Fuling” is:

***Wolfiporia hoelen*** (Fr.) Y.C. Dai & V. Papp, IMA Fungus 12(no. 22): 25 (2021)

*Basionym: Pachyma hoelen* Fr., Syst. Mycol. (Index): 125. 1832, nom. sanct. – Neotype HMAS 248370 (Designated by Wu, Li, Dong, Dai & Papp, Frontiers in Microbiology 11(no. 590,788): 7. 2020).

## 6. Conclusion and recommendations

Based on the recent taxonomic results (Wu et al. 2020), the continuous use of the names *Wolfiporia cocos* and especially *Poria cocos* for the “Fuling” mushroom may cause uncertainty in scientific works and comparing its results. Therefore, in the present work, we recommend the acceptance and use of the taxonomically and nomenclaturally correct names *Pachyma hoelen* or *Wolfiporia hoelen* (Stalpers et al. 2021). Furthermore, in the case of the applied research, we consider it expedient to identify the examined samples based on barcoding sequences and to compare them with the reference sequences of *W. hoelen* published by Wu et al. (2020).
